# Five Fabaceae Karyotype and Phylogenetic Relationship Analysis Based on Oligo-FISH for 5S rDNA and (AG_3_T_3_)_3_

**DOI:** 10.3390/genes13050768

**Published:** 2022-04-26

**Authors:** Zhoujian He, Wei Zhang, Xiaomei Luo, Jie Huan

**Affiliations:** College of Forestry, Sichuan Agricultural University, Huimin Road 211, Wenjiang District, Chengdu 611130, China; hezhouj@163.com (Z.H.); cris_zhang_wei@163.com (W.Z.); 15388135911@163.com (J.H.)

**Keywords:** Oligo-FISH, karyotype, cytogenetics, Fabaceae

## Abstract

Most Fabaceae have nitrogen fixation abilities and are valuable forage and medicinal resources. However, cytogenetic data of many Fabaceae species are unclear. Karyotypes reveal cytological characteristics and are crucial to understanding the organization and evolution of chromosomes in species. Oligo-FISH can reveal genetic composition and karyotype variation patterns with rapid and efficient results. Karyotype analysis of five Fabaceae species by oligonucleotide probes showed that: *Robinia pseudoacacia*, karyotype formula 2n = 2x = 20m + 2sm, cytotype 2B, arm ratio 3.4821, eight chromosomes distributed 5S rDNA signal. The karyotype formula of *Robinia pseudoacacia* ‘idaho’ was 2n = 2x = 20m + 2sm, cytotype 1A, arm ratio 1.8997, and 5S rDNA signal was distributed on six chromosomes. Karyotype of *Robinia pseudoacacia* f. *decaisneana* 2n = 2x = 20m + 2sm, cytotype 1B, arm ratio 2.0787, the distribution of eight chromosomes with 5S rDNA signal. Karyotype formula of *Styphnolobium japonicum* 2n = 2x = 14m + 12sm + 2st, cytotype 2B, arm ratio 2.6847, two chromosomes have 5S rDNA signal. *Amorpha fruticose* karyotype 2n = 2x = 38m + 2sm, cytotype 1B, arm ratio 3.2058, four chromosomes possessed 5S rDNA signal. Both ends of all species’ chromosomes have (AG_3_T_3_)_3_ signals. The results of this study provide chromosome numbers and a physical map, contributing to the construction of the Oligo-FISH barcode and providing molecular cytogenetics data for Fabaceae.

## 1. Introduction

Fabaceae is the third largest flowering plant family [1,2]; because of its ability to form symbiotic relationships with nitrogen-fixing bacteria, some species of this family are commonly used as genetic model organisms [3], with more than 18,000 species [4]. Due to the large number of species, basic research on many species is still lacking.

Compared with other traits, chromosomal traits are always retained in plants [5], and chromosome number and morphology are important components of karyotype [6]. Karyotypes are used to reveal genome organization at the chromosomal level [7], reveal cytological characteristics [8], and provide information on species origin, phylogeny, genetics and breeding, and variety improvement [9,10], and can provide a basis for plant naming [11]. Understanding the development of karyotypes helps to better understand the organization and evolution of chromosomes in related species [12]. Therefore, karyotype analysis has been performed on many Fabaceae plants, such as *Vigna* [13], *Canavalia* [14], *Trigonella* [15], *Hedysarum* [16]. However, the karyotype analysis is more focused on herbaceous and shrub plants than woody plants in Fabaceae. The establishment of chromosome karyotype map is a necessary condition for chromosome research [17]. However, it is often hampered by a lack of markers that allow identification of only individual chromosomes. To this end, many new technologies have been applied to overcome this barrier [14].

Fluorescence in situ hybridization (FISH) is an important tool for plant karyotype construction and has been widely used to map single copy and repeated DNA sequences in plants and to map molecular cytogenetics using chromosomal specific probes [18,19]. FISH signals can show patterns of genetic composition and karyotype variation [20,21]. The combined probes of 5S rDNA and (AG_3_T_3_)_3_ oligonucleotide have been applied to *Hibberus hibberus* [22], *Hippophae rhamnoides* [6], and *Chimonanthus campanulatus* [23]. However, it is the first application of the combined probe in Fabaceae. Two oligonucleotide probes, (AG_3_T_3_)_3_ and 5S rDNA are used to obtain cytogenetic data for five Fabaceae species. The results of this study will be helpful for the identification of chromosomes and the construction of oligonucleotide barcodes in the future.

## 2. Materials and Methods

### 2.1. Seed Materials

All seeds collected from Jiangsu Hengxin Seed Industry Co. LTD. Materials are shown in Table 1. The seeds were soaked in water for 24 h at a temperature of 20 °C and under natural light conditions, then placed in wet filter paper. Approximately 1.5–2.0 cm, root tips were treated with nitrous oxide for 2.5 h. After the treatment, the root tips were placed in glacial acetic acid for 5 min and then maintained in 75% ethyl alcohol at −20 °C until use.

### 2.2. Chromosome Preparation

The ethyl alcohol on the root tips was washed off by ddH_2_O. The root-tip meristems were dispersed by cellulose and pectinase (2:1) and maintained in this mixture at 37 °C for 45 min. After this treatment, the enzyme mixture on the meristems was washed off by using ddH_2_O twice. The ddH_2_O was washed off with ethyl alcohol twice. Subsequently removed all ethyl alcohol. After the meristems were air dried completely, 20 µL of glacial acetic acid was added to each meristem to prepare a suspension, and 10 µL of the mixture was dropped on one clean slide. when slides were air dried, using the Olympus CX23 microscope (Olympus Corporation, Tokyo, Japan) to examine. The metaphase chromosomes of well-spread would be used, the subsequent were used in situ hybridization experiments in the further.

### 2.3. Probe Preparation

The chromosome end 21-bp repeat sequence (AG_3_T_3_)_3_ 5′-AGGGTTTAGGGTTTA GGGTTT-3′ [5], the ribosome 5S rDNA 41-bp sequence 5′-TCAGAACTCCGAAGTTAAGCGTGCTTGGGCGAGAGTAGTAC-3′ [24] Two oligonucleotides (AG_3_T_3_)_3_ [5] and the ribosome 5S rDNA [24] were used in this study. These two probes were tested for the first time in Fabaceae and these oligonucleotide sequences were produced by Sangon Biotechnology Co., Ltd. (Shanghai, China). The probes 5′ ends were labelled with 6-carboxyfluorescein (FAM) or 6-carboxytetramethylrhodamine (TAMRA). The synthetic probes were dissolved in 1 × Tris-Ethylene Diamine Tetraacetic Acid (TE) and stored in maintained at a concentration of 10 µM at −20 ℃.

### 2.4. FISH Hybridization

The slides were fixed in 4% paraformaldehyde for 10 min, immersed in 2 × saline sodium citrate (SSC) buffer for 5 min in twice. After, incubations with 75%, 95%, and 100% ethyl alcohol successively with 5 min. Then, 60 µL of 70% deionized formamide (FA) was dropped the slides, coverslips (24 cm × 50 cm) placed at 80 °C for 2 min. Next step, the coverslips were removed, and slides were then incubated in 75%, 95%, and 100% ethanol (precooled at 20 °C) for 5 min. A total amount of 10 µL hybridization solution, including 1.5 µL of each probe and 8.5 µL mixture of 2 × SSC and 1 × TE, was dropped onto the chromosomes and a cover glass (24 cm × 50 cm). The slides were then incubated at 37 °C for 2 h.

### 2.5. Image Analysis

After hybridization, the slides were shaken and washed with 2 × SSC buffer in order to remove the coverslips. In total, 10 µL of 4,6-diamidino-2-phenylindole (DAPI) was dropped onto the air-dried chromosomes, and coverslips (24 cm × 50 cm) were placed on top of the DAPI solution. The slides were examined using an Olympus BX63 fluorescence microscope combined with a Photometric SenSys Olympus DP70 CCD camera (Olympus Corporation, Tokyo, Japan).

Analysis the signal patterns, by using for three best spreads. Using Photoshop version 2021 (Adobe Systems Inc., San Jose, CA, USA) to calculate the length of each chromosome, and each spread was measured three times, to get an average value. The chromosomes were arranged by length from longest to shortest.

## 3. Results

### 3.1. Karyotype Analysis

**Figure 1:**
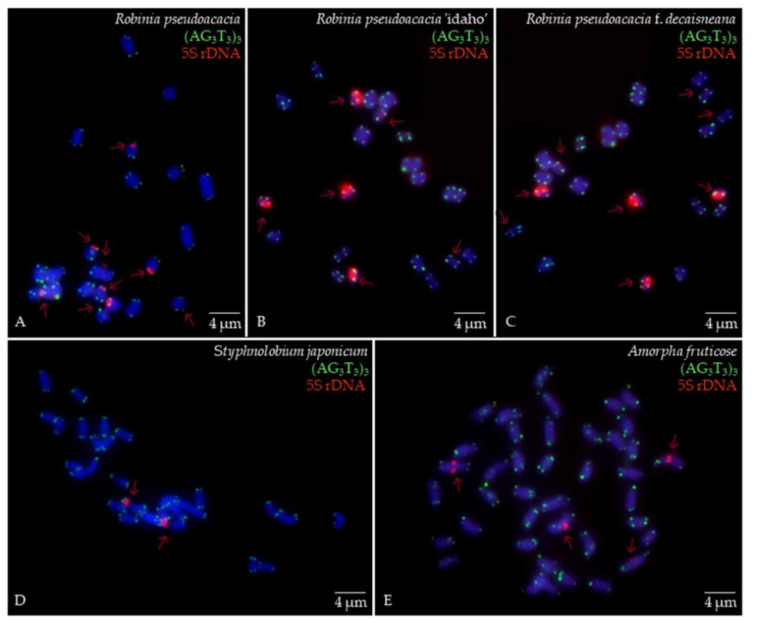
Mitotic metaphase chromosomes fluorescence in situ hybridization (FISH) visualization of *R. pseudoacacia* (**A**), *R. pseudoacacia* ‘idaho’ (**B**), *R. pseudoacacia* f. *decaisneana* (**C**), S. *japonicum* (**D**) and *A. fruticosa* (**E**). The (AG_3_T_3_)_3_ was labelled with 6-carboxyfluorescein (FAM) (green), the 5S rDNA was labelled with 6-carboxytetramethylrhodamine (TAMRA) (red). Five Fabaceae chromosomes were counterstained by 4,6-diamidino-2-phenylindole (DAPI) (blue). Scale bar = 4 µm.

**Figure 2:**
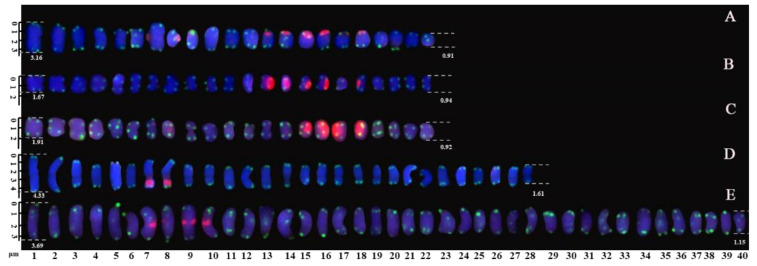
Mitotic chromosomes of five Fabaceae rearranged from Figure 1. (**A**) *R. pseudoacacia*, (**B**) *R. pseudoacacia* ‘idaho’, (**C**) *R. pseudoacacia* f. *decaisneana*. (**D**) *S. japonicum*, and (**E**) *A. fruticose*. According length, the chromosomes were aligned from the longest to the shortest chromosome. The scale bars range from 1 to 4 μm.

**Figure 3:**
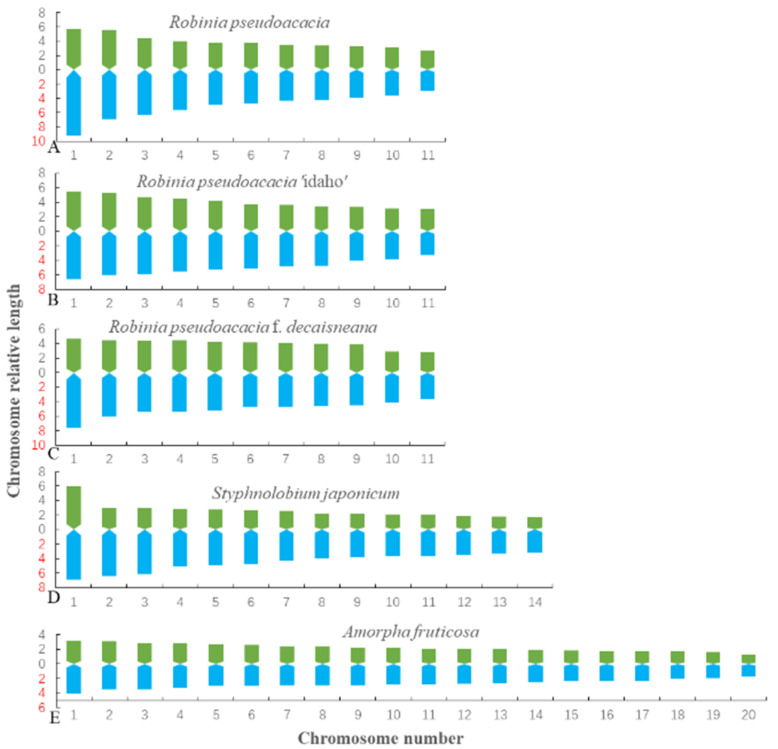
Karyotype ideograph of five Fabaceae. (**A**) *R. pseudoacacia*, (**B**) *R. pseudoacacia* ‘idaho’, (**C**) *R. pseudoacacia* f. *decaisneana*. (**D**) *S. japonicum*, and (**E**) *A. fruticose*. The *x*-axis indicates chromosome number, whereas the *y*-axis indicates relative chromosome length. The data were from Table 1 in order to better display the relative chromosome length of five Fabaceae.

The metaphase chromosomes of five species of Fabaceae were analyzed by FISH, as shown in Figure 1. The chromosome number of *R. pseudoacacia*, *R. pseudoacacia* ‘idaho’ and *R. pseudoacacia* f. *decaisneana* was 2n = 22. The *S. japonicum* chromosome number was 2n = 28. The chromosome number of *A. fruticosa* was 2n = 40. To better characterize the chromosomes of five Fabaceae, Figure 1A–E individual chromosomes were aligned by length from the longest chromosome to the shortest, as illustrated in Figure 2. Karyotype formula for five Fabaceae was shown in Table 2. Chromosomes relative length of five Fabaceae were shown in Figure 3.

### 3.2. Probe Signal Distribution

**Figure 4:**
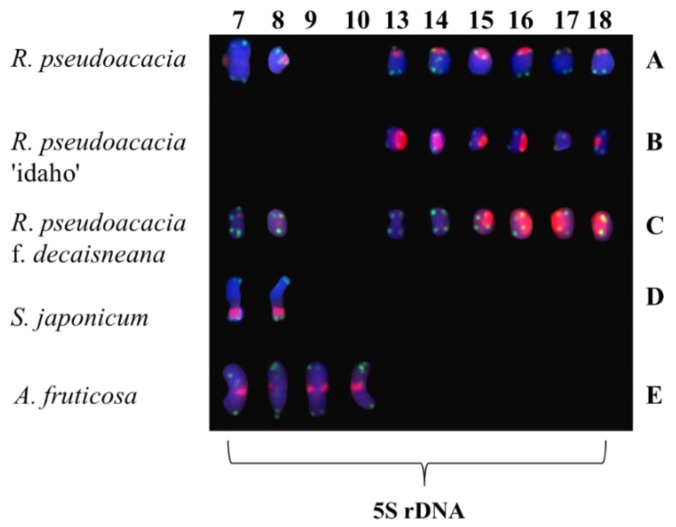
Chromosomes of five Fabaceae identified using 5S rDNA cut Figure 1. (**A**) *R. pseudoacacia*, (**B**) *R. pseudoacacia* ‘idaho’, (**C**) *R. pseudoacacia* f. *decaisneana*. (**D**) *S. japonicum*, and (**E**) *A. fruticose*. The numbers on the upper side represent the chromosome number consistent in Figure 1. Figure 4 is a simplified version of Figure 1.

The (AG_3_T_3_)_3_ signal was present in all chromosomes of five species of Fabaceae, and appeared at the centromere and proximal. The results of (AG_3_T_3_)_3_ signal showed little ability to distinguish the five species of Fabaceae, but it could help to count the chromosome number of the five species of Fabaceae. The 5S rDNA signal was also present in five species of Fabaceae, showing great chromosome discrimination ability. Although *R. pseudoacacia*, and *R. pseudoacacia* f. *decaisneana* each had four pairs 5S rDNA signals, signals locatate were differrnce. *R. pseudoacacia* pairs of 5S rDNA were located in the centromere and three pairs in the telomere or proximal telomere. *R. pseudoacacia* f. *decaisneana* two pairs of 5S rDNA were located in the centromere and two pairs in the telomere or proximal telomere. *R. pseudoacacia* ‘idaho’ had three pairs of 5S rDNA signals, which five chromosomes strong signals and one chromosome weak signal. *S. japonicum* had pairs of 5S rDNA signals located at chromosome proximal telomere. *A. fruticosa* had two pairs of 5S rDNA signals, both located in the centromere. The detailed results are shown in Figure 4.

**Figure 5:**
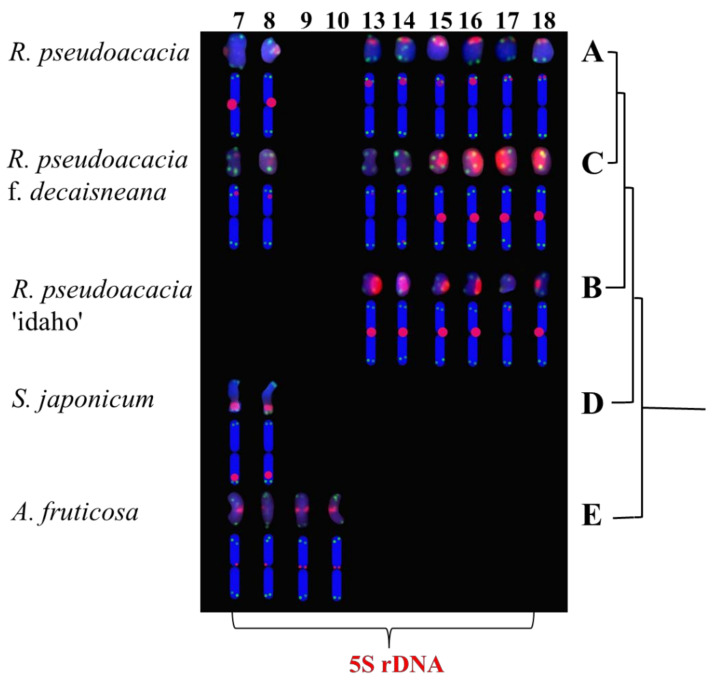
Physical map of Fabaceae. (**A**) *R. pseudoacacia*, (**B**) *R. pseudoacacia* ‘idaho’, (**C**) *R. pseudoacacia* f. *decaisneana*. (**D**) *S. japonicum*, and (**E**) *A. fruticose*. Signal pattern ideographs were constructed based on the signal patterns of the chromosomes mentioned above and the chromosomes in Figure 4. The numbers at the top represent the number of chromosomes, and the 5S rDNA signal type at the bottom were consistent with the five Fabaceae in Figure 4. The blue solid or dotted lines on the right were clustered according to the genetic relationship of 5S rDNA.

5S rDNA signals were used as the main differentiator to determine the genetic relationship. The phylogenetic relationship between *R. pseudoacacia*, and *R. pseudoacacia* f. *decaisneana* was the closest, of which the 5S rDNA had four pairs. These two species were more closely related to *R. pseudoacacia* ‘idaho’ than to *S. japonicum* and *A. fruticose*. *A. fruticose* had two separate 5S rDNA signals; therefore, compared with the other four plants, *A. fruticose* had the most distant relationship (shown in Figure 5).

## 4. Discussion

Chromosome data are useful for plant classification [25] and are fundamental to understanding cytology [26]. As a relatively stable feature, chromosome data is an important means to study chromosome aberrations, cell functions and taxonomic relationships in plant species [5]. The chromosome numbers of *R. pseudoacacia*, *R. pseudoacacia* ‘Idaho’, *R*. *pseudoacacia* f. *decaisneana*, *S. japonicum*, *A*. *fruticose* in this study were consistent with those of previous studies [27,28,29,30,31]. However, the karyotype formula is different. Liu et al. [28] believed that *S. japonicum* 2n = 4x = 28 = 18m + 10sm cytotype 2B, *R.* *pseudoacacia* 2n = 2x = 22 = 4m + 8sm + 10st cytotype 3B and *A. fruticose* 2n = 2x = 40 = 32m + 8sm cytotype 1A, only *R. pseudoacacia* and *A. fruticose* have appendages, whereas the satellite chromosome of *R. pseudoacacia* considered by Chen [27] as short arms. Lv and Wang [30] used the traditional production method and obtained the karyotype formula 2n = 4x = 40= 30m + 8sm + 2st of *A. fruticose*, without satellite, and the cytotype was 2A. Shi et al. [31] obtained the karyotype formula 2n = 2x = 40 = 28m + 8sm + 2st + 2m (SAT) by using three methods of film preparation, and the cytotype was 2B. In this study, no satellite was observed in these species. The reason for the inconsistency between the results and previous studies may be the different methods of film production. Lv and Wang [30] and Shi et al. [31] did not use FISH technique, but used traditional filmmaking techniques, resulting in unclear chromosome images. Liu et al. [28] used FISH technology, but the method was different in the early stage of production, which may lead to insufficient shrinkage of chromosomes. Secondly, one possible reason for the length difference may be the use of different tools to calculate information about chromosome arms and shapes [5]. In addition, which may be due to cell cycle synchronization and low chromosomal diffusion efficiency [32], Or due to the differential accumulation of transposable factors [33,34]. Finally, due to technical reasons, unclear images in previous studies may cause difficulties in measurement, resulting in inconsistent results.

rDNA-FISH signal FISH localization is not only conducive to the identification of chromosomes and the construction of detailed karyotypes, but also can reveal the genome organization of species at the chromosomal level and study the evolutionary relationships of related species [35,36,37,38,39]. The amount and location of 5S and 45S rDNA is often characteristic of a particular species or genus [36,40,41]. FISH has identified the number and location of rDNA loci in over 1,600 plant species [42]. Changes in the number and position of rDNA loci may be related to transposon-mediated transposition, unequal crossover, inversion, translocation and loci replication or deletion [36,43,44,45]. 5S rDNA signaling sites may occur on each auto-chromosome [46], which may occur in the middle, near the middle, or at the end of the chromosome [5].

5S rDNA oligonucleotide probes have been widely used in Fabaceae, for example: *Vigna* [13], *Hedysarum* [16], *Canavalia* [14], *Phaseolus* [17]. However, 5S rDNA is mostly used in Fabaceae herbs and lianas, but rarely reported in woody plants. These 5S rDNA signals vary in location and intensity, and can be used for species ploidy identification, as well as for intraspecific and interspecific species identification and phylogenetic relationship identification [6,22,47,48]. *R. pseudoacacia* and *R. pseudoacacia* f. *decaisneana* both had four pairs of 5S rDNA signals, indicating that they were most closely related. *R.* *pseudoacacia* ‘idaho’ has three pairs of 5S rDNA signal and is closely related to *R.* *pseudoacacia* and *R. pseudoacacia* f. *decaisneana*. *A. fruticosa* showed the 5S rDNA signal for pairs of new chromosomes, which was not found in the other four species; therefore, it was the most distant relationship.

Telomeres exist at the natural ends of linear chromosomes in eukaryotes and are structurally and functionally distinct from other DNA sequences [49]. (AG_3_T_3_)_3_ Telomere probes have not been used in Fabaceae in the past, but have been used in *Berberis diaphana* [50], *Hibberus hibberus* [22], *Hippophae rhamnoides* [6], and *Chimonanthus campanulatus* [23]. (AG_3_T_3_)_3_ probes are usually distributed at both ends of chromosomes to determine the integrity of chromosomes and count the number of chromosomes [22] In this study, all Fabaceae species showed the (AG_3_T_3_)_3_ signal, which was beneficial to chromosome count, and the results were consistent with previous studies. Further research suggests that the true diversity of telomere sequences in terrestrial plants may have been underestimated and requires further study [51].

In this study, the five materials could be distinguished by 5S rDNA signal, but the combination of two probes could not be used to map chromosomes and distinguish each chromosome. It is also impossible to map oligonucleotide barcodes to study the chromosomal evolutionary relationships among related species. In the future, we will develop more oligonucleotide probes to study the evolutionary relationships of chromosomes.

## 5. Conclusions

The result of this study is that (AG_3_T_3_)_3_ and 5S rDNA can effectively distinguish five Fabaceae species by signals. This provides a chromosome number and a physical map, contributing to providing molecular cytogenetics data for Fabaceae.

## Figures and Tables

**Table 1:** Collection of seed resources.

NO.	Name	Location	Type
1	*Robinia pseudoacacia* L.	Jiangsu, China	seed
2	*Robinia pseudoacacia* ‘idaho’	Jiangsu, China	seed
3	*Robinia pseudoacacia* f. *decaisneana* (Carr.) Voss	Jiangsu, China	seed
4	*Styphnolobium japonicum* (L.) Schott	Jiangsu, China	seed
5	*Amorpha fruticose* L.	Jiangsu, China	seed

**Table 2:** Karyotype analysis of five Fabaceae. Relative chromosome length of five Fabaceae was omitted. Karyotype asymmetry index, according to Stebbins (1971).

Species	Karyotype	Cytotype	Arm Ratio
*R. pseudoacacia*	2n = 2x = 20m + 2sm	2B	3.4821
*R. pseudoacacia* ‘idaho’	2n = 2x = 20m + 2sm	1A	1.8997
*R. pseudoacacia* f. *decaisneana*	2n = 2x = 20m + 2sm	1B	2.0787
*S. japonicum*	2n = 2x = 14m + 12sm + 2st	2B	2.6847
*A. fruticosa*	2n = 2x = 38m + 2sm	1B	3.2058

## Data Availability

All data and materials are included in the form of graphs in this article.
